# Association between Asymptomatic Bacteriuria and Pre-Eclampsia

**DOI:** 10.5539/gjhs.v8n7p235

**Published:** 2015-12-16

**Authors:** Negin Rezavand, Firooze Veisi, Mrayam Zangane, Roghaye Amini, Afshin Almasi

**Affiliations:** 1 High Risk Pregnancy Research Center, Kermanshah University of Medical Sciences, Kermanshah, Iran

**Keywords:** asymptomatic bacteriuria, pre-eclampsia, pregnancy

## Abstract

Asymptomatic bacteriuria is one of the most common and important bacterial infections during pregnancy and can result in progressive infections and endanger maternal as well as fetal health. In this study, we assessed the relationship between asymptomatic bacteriuria and pre-eclampsia. In this case-control study, pregnant women who presented to Imam Reza Hospital in Kermanshah in 2013-14 were studied. The minimum sample size was calculated as 125 pregnant women in each group with a total of 250 subjects. There were 125 women with pre-eclampsia and 125 women without pre-eclampsia (control group). Matching was done for age, gestational age, and parity between case and control groups. Matching was verified by a P value of 0.061 for maternal age and gestational age and 0.77 for parity. The statistical analyses were done by applying the chi-squared test and determining odds ratio (OR) for having bacteriuria in univariate logistic regression as well as multivariate regression with adjusting the effect of maternal age, gestational age, and parity. Pyuria and bacteriuria were significantly more common in pre-eclampsia group than in control group. The results showed that a significant association existed between asymptomatic bacteriuria and pre-eclampsia. The rate of asymptomatic bacteriuria was 6.8 times higher in women with pre-eclampsia compared to those without pre-eclampsia. Further studies are required for better clarification of association between asymptomatic bacteriuria and pre-eclampsia.

## 1. Introduction

Asymptomatic bacteriuria is one of the most common and important infections during pregnancy which can cause progressive and severe infections and endanger maternal as well as fetal health. Bacteriuria is usually associated with low birth weight, high blood pressure during pregnancy, maternal anemia, and fetal death (Cekmen et al., 2003; Graham, Leathart, Keegan, Pearson, Bint, & Gally, 2001; Acharya et al., 2013).

Asymptomatic bacteriuria is characterized by continuous growth and activity of bacteria inside the urinary tract of women who are asymptomatic. The incidence rate of this condition during pregnancy varies from 2-7 per cent. In fact, urinalysis of such patients demonstrates considerable bacteriuria without pyuria. Urine culture also becomes positive (American Academy of Pediatrics and American College of Obstetricians and Gynecologists, 2012; Chaemsaithony et al., 2013). Studies performed in recent years in identifying factors responsible for pre-eclampsia showed that primary infections during pregnancy increase the chance of pre-eclampsia (Sheffield & Cunningham, 2005; Chang, Armstrong, Ebeling, Hulsey, & Newman, 2006).

Pre-eclampsia is a specific syndrome during pregnancy with prevalence ranging from 5 to 7%. This is an important cause of maternal and fetal morbidity and mortality. Pre-eclampsia can adversely affect all body systems and is defined by blood pressure of greater than 140/90 mmHg after the 20^th^ week of pregnancy and proteinuria of equal to or higher than 300 mg per 24 hours. By affecting all maternal body systems, pre-eclampsia predisposes the mother to high-risk pregnancy and can result in hazardous outcomes for both the mother and her fetus (Cor, 2006; Hazhir, 2007; MCI Saac et al., 2005). Despite extensive studies about recognizing the etiology of pre-eclampsia and its high mortality, all mechanisms of endothelial dysfunction and resultant pre-eclampsia have not been described. Studies are ongoing to find clinical markers to predict and prevent development of pre-eclampsia (Turan et al., 2008).

It is likely that subclinical infections result in increased maternal cytokines and subsequently cause pre-eclampsia via affecting the vascular endothelium (Boroumand, Sam, Abbasi, Salarifeur, Kassaian, & Forghani, 2006; Gurrieri et al., 2012).

Since asymptomatic bacteriuria is one of the most common conditions during pregnancy and can have adverse effects on pregnancy, this study was performed to investigate the relationship between asymptomatic bacteriuria and development of pre-eclampsia in hospitalized patients.

## 2. Materials and Methods

In this study which lasted from 2013 to 2014 at Imam Reza Hospital of Kermanshah, Iran, two groups of pregnant women were included. One group consisted of 125 women with pre-eclampsia and the other group was control group without pre-eclampsia (125 pregnant women). All women were at 28 weeks of gestation or later and all of them had singleton pregnancy. The inclusion criteria included lack of systemic or infectious diseases or taking antibiotics in the last three months. The informed consent was obtained and the subjects were reassured that their information will be kept confidential and no cost will be charged during the study.

After the inclusion, the mothers were trained how to accurately collect clean mid-stream urine samples. Urinalysis and urine culture were performed for the subjects. Pyuria was defined as ≥ 5 white blood cells per high power field (hpf) after urine centrifuging. Bacteriuria was defined as presence of any number of bacteria in the sample. Positive urine culture was defined as colony of one type of bacteria for more than 10^5^ bacteria in mL of the urine sample.

The two groups were matched regarding maternal age and parity. The diagnostic criteria for pre-eclampsia were blood pressure of ≥ 140/90 mmHg and proteinuria of ≥ +1 or more than 300 mg per 24 hours. The data were entered into a checklist and were analyzed by SPSS software (ver. 20.0). The statistical tests used were chi-squared test and calculating odds ratio (OR) via regression analysis for having bacteriuria.

## 3. Results

Mean age of pre-eclampsia group was 29.68 years and in control group it was 28.30 years with no significant difference (independent t-test, P value = 0.61). Mean gestational age in pre-eclampsia group was 34.04 years and in control group it was 33.82 with no significant difference (independent t-test, P value = 0.64).

Regarding parity, the chi-squared test showed a P value of 0.77 and matching was confirmed (Table 1).

**Table 1:** Frequency distribution (percentage) of 250 pregnant women based on parity in pre-eclampsia and control groups

Study groups	Frequency distribution	Parity			Total

		1	2	≥3	
**Control**	Frequency	54	41	30	125
	Percentage	43.2%	32.8%	24%	100
**Pre-eclampsia**	Frequency	49	42	34	125
	Percentage	39.2%	33.6%	27.2%	100
**Total**	Frequency	103	83	64	250
	Percentage	41.2%	33.2%	25.6%	100

Mean systolic and diastolic blood pressures showed a significant difference between the two groups (P < 0.001); Table 2.

**Table 2:** Mean (standard deviation) of systolic and diastolic blood pressure in the two groups

Variable	Group	Mean	Standard deviation	P value
**Systolic blood**	Pre-eclampsia	148.2	10.8	< 0.001
**pressure**	Control	107.16	7.5	
**Diastolic blood pressure**	Pre-eclampsia	96.08	5.66	< 0.001
	Control	68.32	6.8	

In pre-eclampsia group, 69 subjects (55.2%) had pyuria. This figure was 28 cases (22.4%) in control group. The odds of having pyuria in pre-eclampsia group was 4.2 times higher than in control group based on chi-square result (28.33) with 95% confidence interval of 2.38 to 7.69. There was a significant difference regarding asymptomatic bacteriuria with positive urine culture between pre-eclampsia group (58 cases, 46.4%) and control group (14 cases, 11.2%); chi-square = 37.3. The odds of having positive urine culture were 6.8 times higher in pre-eclampsia group (P< 0.001).

## 4. Discussion

The reported asymptomatic bacteriuria in pregnant women has been noted as 13.1%, 8.9%, and 10.1% respectively in Zahedan, Sanandaj, and Tehran, Iran (Danesh Shahraki, Pishva, Mirbaha & Arabzadeh, 2010; Enayat & Fariba Bahram, 2008). This rate has been reported as 14.2% in Saudi Arabia, 28.5% in Pakistan, 23.9% in Nigeria, 4% in Australia, and 2.2% in London (Hazhir, 2007; Daneshyar, Mosavibahar, & Alikhani, 2010; Tadesse, Negash, & Ketema, 2007).

These figures reflect variable rates of asymptomatic bacteriuria in different geographic regions. It seem that various factors such as age, sexual activity, socioeconomic status, parity, history of urinary tract infection before pregnancy, anatomic malformations of the urinary system, and gestational age all have influential role in asymptomatic bacteriuria rate. With advancing gestational age, especially in the third trimester, the rate of bacteriuria increases.

In our study, the rate of asymptomatic bacteriuria was significantly higher in pre-eclampsia group than in control group. For the first time in 1936, Peters et al. suggested association between bacteriuria and pre-eclampsia (Peters, Lilvietes, & Zimmerman, 1936). Then, Smith and Bullen reported that bacteriuria was more common in pregnant women with pre-eclampsia compared to those without pre-eclampsia (Kincaid & Bullen, 1965). Stuart et al. later noted that pre-eclampsia was 4 times more common in women with bacteriuria than in those without bacteriuria (Stuart, Cummins, & Chin, 1965).

The studies by Borghei in Gorgan, Akerele, and Caroline showed similar results as ours. They reported that bacteriuria was significantly more common in pregnant women with pre-eclampsia (Bourghei, Kashani, & Rabiei, 2004; Akerele, Abhulimen, & Okonofua, 2001; Caroline, Sara, David, Oona, & Liam, 2013).

In studies by Shamsi in Pakistan and Rizek from UAE, no significant difference was seen regarding bacteriuria between pre-eclampsia group and control group (Shamsi, Hatcher, Shamsi, Zaberi, & Qadri Saleem, 2010; Rizk, Mustafa, & Thomas, 2001).

Maybe the agreement of our results with those of reported by Caroline, Borghei, and Akerele is the adjustment for confounding variables such as gestational age, maternal age, and parity with matching. The discrepancy with the results by Shamsi and Rizek may be due to differences in cultural and socioeconomic status and the fact that the mentioned studies did not adjust confounding variables. The high rate of asymptomatic bacteriuria observed in the current study can be the result of the method of sampling and cultural and social status of the study population. Also, gestational age of pre-eclampsia group was averagely 34.4 weeks and it was 33.82 weeks in control group and all the studied women were in their third trimester. The likelihood of asymptomatic bacteriuria is the highest in the third trimester. A suggested theory is that infection can cause raised maternal serum cytokines. These cytokines damage endothelial function. The possibility of pre-eclampsia is the highest when there is previous history of contact with antigens that affect the body and especially affect the lymphocytes function (Herrera, Chaudhuri, & Lopez, 2001; Tinna Korn, Maria, Jerrie, Jimmy, Jun, & Susan, 2002; HSU & Witter, 1995).

In general, urinary system infections are more common in women with pre-eclampsia and this may reflect a background disease in the kidneys. The likelihood of mild and severe pre-eclampsia in women with only one episode of urinary tract infection was 1.3 and 1.8 times higher compared to those without history of urinary tract infection, respectively (Karmon & Sheiner, 2008).

## 5. Conclusion

Regarding the obtained findings, asymptomatic bacteriuria was a common condition among pregnant women of our study. The complications of this condition can be hazardous for mother and her fetus, although further studies with larger sample size and with control of confounding factors are suggested. However, it seems necessary to screen mothers with urine culture and accurate microscopic methods including sampling for two times for better diagnosis of bacteriuria and its complications in the third trimester.

## References

[ref1] Acharya A, Santos J, linde B, Anis K (2013). Acute kidney injury in pregnancy current status. Adr chronic kidney Dis.

[ref2] Akerele J, Abhulimen P, Okonofua F (2001). Prevalence of asymptomatic bacteriuria among pregnant women in Benin City. J obstet Gynecology.

[ref3] American Academy of pediatrics and American college of obstetricians and Gyn ecologists (2012). Guidelines for perinatal care.

[ref4] Boroumand M, Sam L, Abbasi S, Salarifeur M, Kassaian E, Forghani S (2006). A symptomatic bacteriuria in type 2 Iranian diabetic women:a cross Sectional Study. BMC Womens Health.

[ref5] Bourghei N, Kashani E, Rabiei M (2004). The relation between Asymptomatic bacteriuria and preeclampsia. J Gorgan uni med Sci.

[ref6] Caroline M, Sara L, David J, Oona C, Liam S (2013). Acute maternal infection and risk of preeclampsia: A population-Based case-control study. Plos One.

[ref7] Cekmen B. M, Erbagci A. B, Balat A, Duman C, Maral H, Ergen K, Kuskay S (2003). Plasma lipid and lipoprotein concentrations in pregnancy-Induced hypertention. Clin Biochem.

[ref8] Chaemsaithony P, Romero R, Korzeniewski S. J, Schwartz A. G, Stampalija T, Dong Z, Chaiworapongsa T (2013). Soluble trail in normal pregnancy and acute pyelonephritis:a potential explanation for the susceptibility of pregnant women to microbial products and infection. The journal of maternal-fetal & neonatal medicine:the official journal of the European Association of Perinatal Medicine, the Federation of Asia and Oceania Perinatal Societies, the International Society of Perinatal Obstetricians.

[ref9] Chang F, Armstrong D, Ebeling M, Hulsey I, Newman R (2006). Urinary tract infections are associated with an increased risk of preeclampsia. Am J Obstetrics & Gyn Ecol.

[ref10] Cor J (2006). Urinary tract infections in women: Diagnosis and management in primary care. BMJ.

[ref11] Danesh Shahraki A, Pishva A, Mirbaha S, Arabzadeh A (2010). The Prevalence of Asymptomatic Bacteruria in pregnant Women with and Without Gestational Diabets. Journal of Isfahan Medical School.

[ref12] Daneshyar E, Mosavibahar S. H, Alikhani M. Y (2010). Association between Asymptomatic Bacteriuria and Some Emographic Variables in Pregenant Women Refered to health centers Affiliated to Hamedan University of Medical Sciences. Scientific Journal of Ilam University of Medical Sciences.

[ref13] Enayat K, Fariba F, Bahram N (2008). Asymptomatic bactriuria among pregnant women referred to outpatient clinics in sanandaj, Iran. Int Braz J Urol.

[ref14] Graham J, leathart J, Keegan S, pearson J, Bint A, Gally D (2001). Analysis of Escherchia coli strain Causing bactariuria during pregnancy:selection for strains that do not express type I fimberiae. Infect Immun.

[ref15] Gurrieri C, Garovic V. D, Gullo A, Bojanić K, Sprung J, Narr B. J, Weingarten T. N (2012). Kidney injury during pregnancy:associated comorbid conditions and outcomes. Archives of Gynecology.

[ref16] Hazhir S (2007). Asymptomatic bacteriuria in pregnant women. Urol J.

[ref17] Herrera J, Chaudhuri G, Lopez P (2001). Is infection a major risk factor for preeclampsia?. Medical Hypothesis.

[ref18] Hsu C, Witter F (1995). Urogenital infection in preeclampsia. Inter J Gynecol & Obstet.

[ref19] Karmon A, Sheiner E (2008). The relationship between urinary tract infection during pregnancy and preeclampsia. Arch Gynecol Obstet.

[ref20] Kincaid P, Bullen M (1965). Bacteriuria in pregnancy. Lancet.

[ref21] MCI Saac W, Carroll J, Biringer A. B, Ernstein P, Lyons E, Low D (2005). Screeing for asymptomatic bacteriuria in pregnancy. J Obstet Gynaccol.

[ref22] Peters J, Lilvietes P, Zimmerman H (1936). Pyelitis in toxemias of pregnancy. Am J obstet Gyn ecd.

[ref23] Rizk D, Mustafa N, Thomas L (2001). The prevalence of urinary tract infections with gestational diabetes mellitus. Int Urogynecol J Pelvic Floor Dysfun.

[ref24] Shamsi U, Hatcher H, Shamsi A, Zaberi N, Qadri Saleem S. A (2010). Multicenter matched case control study of risk factors for preeclampsia in healthy women in Pakistan. BMC Women's Health.

[ref25] Sheffield J, Cunningham F (2005). Urinary tract infection in women. Obstetrics & Cynecd.

[ref26] Stuart K, Cummins G, Chin A (1965). Bacteriuria, prematurity, and the hypertensive disorders of pregnancy. Br med J.

[ref27] Tadesse A, Negash M, Ketema L (2007). Asymptomatic bacteriuria in pregnancy: Assessment of prevlence microbial agents and there antimicrobial sensitivity pattern in condar teaching hospital, North West Ethiopia. Ethiop Med J.

[ref28] Tinna Korn C, Maria T, Jerrie R, Jimmy E, Jun Y, Susan B (2002). Maternal lymphocyte Subpopulations in preeclampsia. AM J Obstet Gynecol.

[ref29] Turan H, Serefhanoglu K, Torun A, Kulaksizoglu S, Kulaksizolu M, Pamuk B (2008). Frequency, risk factors, and responsible pathogenic microorganisms of asymptomatic bacteriuria in patients with type 2 diabetes mellitus. J pn J Infect Dis.

